# Short-term clinical outcomes of primary total knee arthroplasty with a new-type kinematic retaining implant: A comparison with preexisting cruciate retaining prosthesis

**DOI:** 10.1097/MD.0000000000034769

**Published:** 2023-08-25

**Authors:** Takashige Momose, Masaki Nakano, Yukio Nakamura, Takashi Maeda, Atsushi Sobajima, Susumu Morioka, Masashi Nawata

**Affiliations:** a Department of Orthopaedic Surgery, Marunouchi Hospital, Matsumoto, Nagano, Japan; b Department of Orthopaedic Surgery, Shinshu University School of Medicine, Matsumoto, Nagano, Japan; c Department of Orthopaedic Surgery, Aizawa Hospital, Matsumoto, Nagano, Japan.

**Keywords:** clinical outcomes, cruciate retaining implant, kinematic retaining implant, patient satisfaction, total knee arthroplasty

## Abstract

Despite the success of total knee arthroplasty (TKA), current implant designs could not consistently restore the physiological knee kinematics, especially in cruciate-retaining (CR) implants. This study aimed to investigate the short-term clinical outcomes, particularly patient satisfaction, of primary TKA employing a new-type kinematic retaining (KR) implant. We analyzed 149 cases applied the KR implant at our institutions during June 2017 to May 2019. The effectiveness of this implant design was compared with another CR one (171 cases). Both groups underwent primary TKA in the same period and all patients completed 2 years of follow-up. Perioperative changes in range of motion (ROM), Knee Score, function score, and patient satisfaction by Forgotten Joint Score-12 (FJS-12) method were evaluated. Postoperative ROM, Knee Score, and function score were significantly improved at 1 year after surgeries and maintained for another year in both KR and CR groups. The improvement rate of ROM in KR group (108.1%) was substantially higher than that in CR (104.5%), even 4% increase could have affected patients’ satisfaction in a real-world setting. Regarding the patient satisfaction, such 4 items as climbing stairs, walking on a bumpy road, doing housework or gardening, and taking a walk or hiking were significantly enhanced in KR cases compared to CR. There were no loosening or revision cases and the short-term survivorships of both implants were 100%. In addition, there has been no case of obvious complications in both groups during and after surgeries. The results of the present study suggest that this novel KR prosthesis can reproduce physiological knee kinematics, recover its functions, and contribute to pain relief after TKA. TKA procedure using the KR implant should be a good surgical option to improve postoperative outcomes.

## 1. Introduction

The number of total knee arthroplasty (TKA) procedure has been increasing worldwide with a predicted future increase of 143% by 2050.^[1]^ So far, good clinical results have been reported in the literature with a survivorship of primary TKA ranged between 90% and 95% in 15-year follow-up duration.^[2]^ Given the increasing number of patients at a higher risk of TKA failure,^[3]^ the increase in revision TKA incidence is not a surprising matter.^[4]^ Unfortunately, the survival rate of revision TKA is reportedly inferior to primary TKA, ranging from 71% to 86% at 10 years of follow-up.^[2]^

Nearly 14% to 39% of TKA patients have reported dissatisfaction due to an incomplete recovery of functions; thus, the efforts to obtain normal knee kinematics may improve function and satisfaction of patients after TKA.^[5]^ Despite the success of TKA procedures, modern implant designs may not be able to consistently restore the physiological knee kinematics and that could be a factor for patients’ dissatisfaction following surgeries. While achieving neutral mechanical alignment is an important element for stability and longevity of TKA, it remains an unresolved issue in the current TKA implant designs.

It has been expected that the new-type kinematic retaining (KR) prosthesis holds excellent clinical outcomes in range of motion (ROM) acquisition, pain reduction, and functional improvement; because that is a new type of implant with a shape which mimics healthy knee. This KR implant is designed to preserve posterior cruciate ligament and consists of asymmetric articular surfaces with a concave medial shape and a saddle-like lateral shape to reproduce the natural knee joint kinematics. It can induce the physiological movement of roll-back and rotation of the femur over the tibia. The prosthesis has been developed to restore physiological kinematics and promote rapid functional recovery, pain relief, and good implant stability. The multiple radii (J-curve) of the femoral condyles vary from medial side to lateral side, allowing the femur to physiologically tension the collateral ligaments during the flexion and extension cycles. Thinner anterior flange and large deep trochlear groove permit an optimal patellar tracking and patellar tilt angle, optimizing the quadriceps performance. The geometry and interaction between tibial and femoral articular surfaces allow posterior femoral roll-back and axial rotation, thus enable the reduction of sliding friction in early-flexion, balancing of soft tissue, and prevention of mid-flexion instability.^[6]^

To the best of our knowledge, there have been few reports on the clinical results of this KR implant, worldwide. This study aimed to investigate the 2-year clinical outcomes, especially patient satisfaction, and compare this new type of prosthesis, which have human knee-imitated asymmetric lateral convex tibial surface, with a traditional bi-concave surfaced implant by employing Forgotten Joint Score-12 (FJS-12) method.^[7]^ We hypothesized that this new-type KR implant might show better results on patient satisfaction etc owing to its kinematic structure.

## 2. Methods

### 2.1. Study subjects

Since the introduction of the KR prosthesis into our country in June 2017, we have performed TKA employing this implant in more than 180 knees of 160 patients to date. We examined and evaluated the short-term clinical results and postoperative patient satisfaction of primary TKA in 149 cases (42 male and 107 female, 164 knees) applied Physica KR^®^ (Lima Corporate, Villanova, San Daniele del Friuli, Udine, Italy. Supplemental Figure 1, http://links.lww.com/MD/J569), which was launched in 2015, at our institutions during June 2017 to May 2019. The navigation system was not used in the present study subjects. The average ± standard deviation age of patients at operation was 70.9 ± 7.5 years, and the body mass index was 25.2 ± 3.7. The inclusion criteria were primary knee osteoarthritis (OA) and rheumatoid arthritis (RA) patients without complications or with minor one(s), such as hypertension and diabetes. The patients using steroid drugs were excluded. One hundred forty-five patients suffered from end-stage OA and remaining 4 were RA patients.

In this study, we validated the effectiveness of the KR design and compared it with preexisting another cruciate retaining (CR) prosthesis, Persona CR^®^ (Zimmer Biomet, Warsaw, IN, USA. Supplemental Figure 1, http://links.lww.com/MD/J569). Implant selection had been done based on the evaluation by 4 different certified joint surgeons. The number of cases applied this CR implant was 171 (49 male and 122 female, 188 knees), and the mean ± standard deviation age and body mass index of patients were 71.2 ± 7.7 years and 25.0 ± 3.6, respectively. The patients suffered from OA (n = 164) and RA (n = 7). Both KR and CR groups underwent primary TKA procedure in the same period; thus, this study was not a case-control one. Preoperative ligament laxity of the knee joints in each patient was not significantly obtained since no medial collateral ligaments were reconstructed in all subjects. This investigation included all cases of primary TKA employing the KR and CR implants performed at our institutions between June 2017 and May 2019. All of the cases showed grade III or IV according to the Kellgren–Lawrence classification preoperatively. On the other hand, in most cases, patella was not resurfaced since patella surface inside and patella-femoral joint were well maintained (less than grade II according to the Kellgren–Lawrence classification), but patella was resurfaced due to the deterioration of joint surface in some cases.

All patients completed the 2 years of follow-up. Following postoperative physiotherapies for almost 1 month during hospitalization in all cases and for 1 to 3 months during clinical outpatient visits in each patient were practiced: ROM exercise, quadriceps training, and activities of daily living in order to improve knee function such as walking. The rehabilitation goal after surgery was more than 100% of postoperative ROM compared to preoperative value and walking without cane. There were no statistically significant differences in the preoperative attributes and scores between the groups (Table 1).

**Table 1:** Patient background data.

	KR group	CR group	*P* value
Cases (knees)	149 (164)	171 (188)	
Gender, male/female	42/107	49/122	1.00^a^
Age (yr)	70.9 ± 7.5	71.2 ± 7.7	.64^b^
BMI, kg/m^2^	25.2 ± 3.7	25.0 ± 3.6	.37^b^
Preoperative ROM, degrees	124.1 ± 8.7	125.4 ± 9.3	.58^c^
Preoperative KS, points (0–100)	51.9 ± 10.6	52.5 ± 11.1	.61^c^
Preoperative FS, points (0–100)	59.4 ± 20.4	63.4 ± 21.2	.17^c^

Mean ± standard deviation values are presented at age, BMI, and preoperative ROM, KS, and FS.

BMI = body mass index, CR = cruciate retaining, FS = function score, KR = kinematic retaining, KS = Knee Score, ROM = range of motion.

a Chi-squared test.

b Student’s *t* test.

c Wilcoxon rank-sum test.

### 2.2. Surgical procedures

Surgical implant position was basically planned preoperatively in all cases using 3-dimensional (3D) templates with the goal of mechanically aligned placement. Based on the determined femoral rotational angle by 3D templates, femoral cutting was performed parallel to surgical transepicondylar axis line during surgeries as a reference axis. After tibial cutting, gap measurements were made to assess the insert thickness, and then the rotation angle of tibial component was determined by using our ROM technique in the KR prosthesis. The KR implant consists of asymmetric articular surfaces with a concave medial shape and a convex lateral shape; therefore, lateral side of femoral component should be placed ahead of the convex lateral shape when knee joint is extended. This is the reason why ROM technique was used to determine the rotation angle of tibial component. It is highly possible that if Akagi line^[8]^ was used in the KR implant, convex at insert surface and convex at femoral component in knee extension position may collide to each other.

On the other hand, the angle of tibial component was assigned based on Akagi line in the CR prosthesis. Since the CR implant consists of symmetric articular surfaces with bi-concave medial and lateral shapes, tibial cutting surface is likely matched on the articular surface. Thus, Akagi line is ideal for the rotation angle of tibial component. In addition, tibial cutting was routinely performed reducing 3° as posterior tilt, which is defined as tibial slope, compared with preoperative original tibial shape. Another point during surgery was a rotational position determination of the tibial component after confirming that the distal end of femoral component matched anterior part of lateral convex surface of insert employing ROM technique. Postoperative plain radiograph was performed and confirmed regarding femoral and tibial implant placement in all cases.

### 2.3. Outcome evaluations

ROM (0° as full extension) examined by direct vision, improvement rate, Knee Score, function score, and FJS-12, which is a patient-reported outcome questionnaire of joint awareness, as well as changes in plain radiographs over time and presence or absence of revision case were evaluated. Improvement rates of ROM were calculated, on which 100% was a baseline in each patient. Postoperative clinical results and patient-reported outcomes by KR implant were compared with those by CR. The significance of differences among each time point was evaluated by Kruskal–Wallis testing, while that between the groups was assessed by Wilcoxon rank-sum test. A 2-tailed *P* value of <.05 was considered significantly different.

### 2.4. Ethical approval

This study was approved by the Institutional Review Board of Marunouchi Hospital, Japan, prior to its commencement. The research procedure was conducted in accordance with the ethical guidelines of the 2013 Declaration of Helsinki. Written informed consent for the research and publication was obtained from all subjects enrolled in this study.

## 3. Results

**Figure 1.**
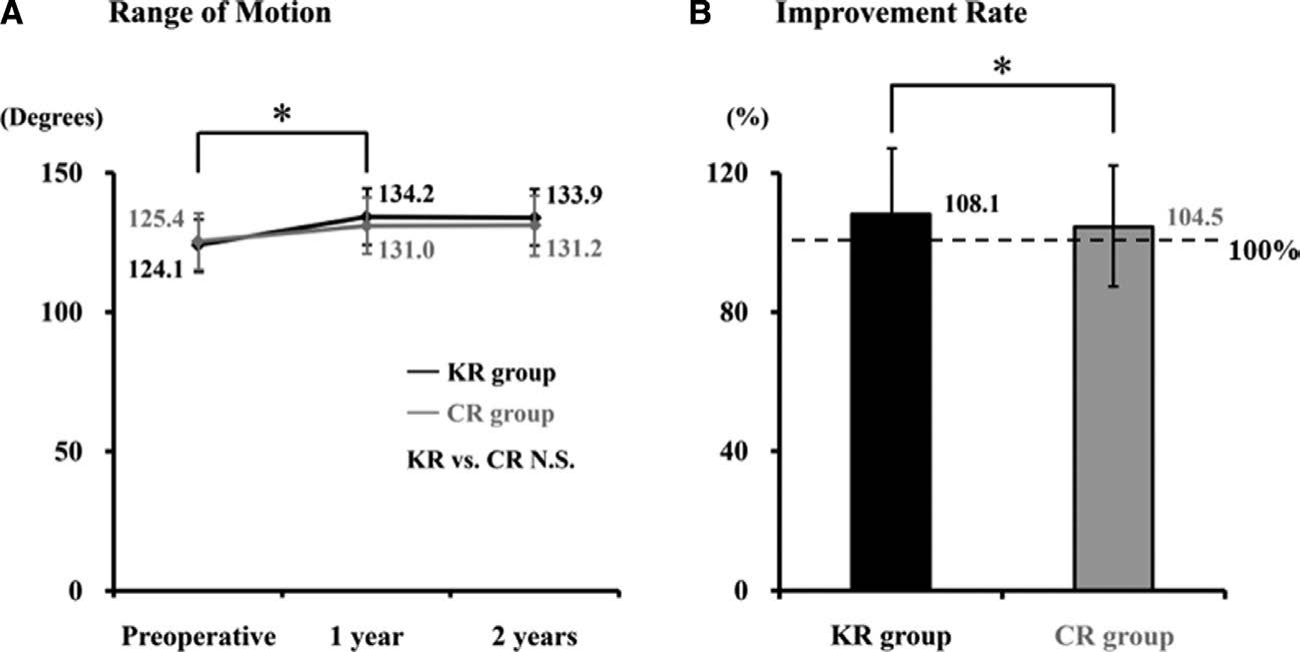
Perioperative range of motion (ROM) examination. (A) Changes and (B) improvement rates of ROM are shown. Significance of differences was evaluated by Kruskal–Wallis test or Wilcoxon rank-sum test (*P < .05). NS = not significant.

**Figure 2.**
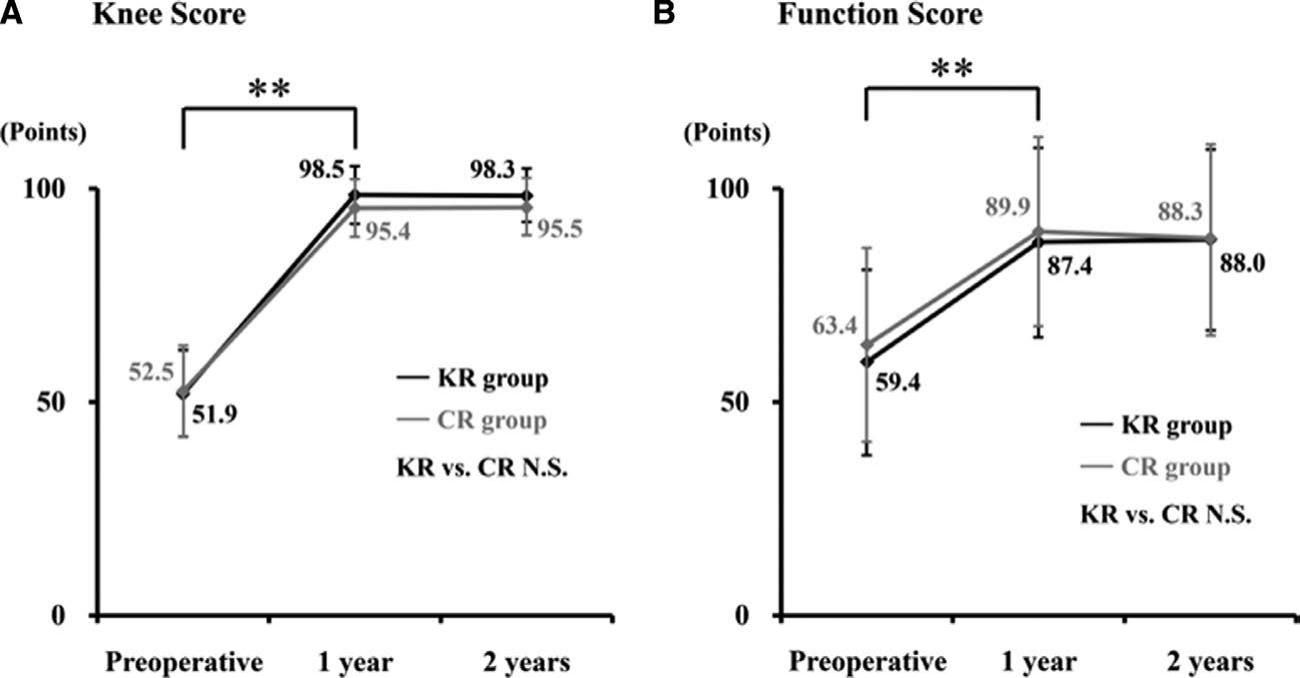
Perioperative Knee Score (KS) and function score (FS) assessment. Changes in (A) KS and (B) FS are shown. Significance of differences was evaluated by Kruskal–Wallis test or Wilcoxon rank-sum test (**P < .01). NS = not significant.

There were no significant disparities in operative time and blood loss between 2 procedures. Postoperative ROM was significantly improved in both KR and CR groups at 1 year afterward, and the improvement was maintained for at least 2 years after surgery (Fig. 1A). Although there were no significant differences in ROM values between the groups, the improvement rate of KR group (108.1%) was substantially higher than that of CR group (104.5%) postoperatively, as the preoperative value was 100% (Fig. 1B). As for the other clinical outcomes, Knee Score and function score of KR group showed significant enhancements at 1 year postoperatively and sustained for another year. The scores were both comparable with CR group (Fig. 2A and B).

**Figure 3.**
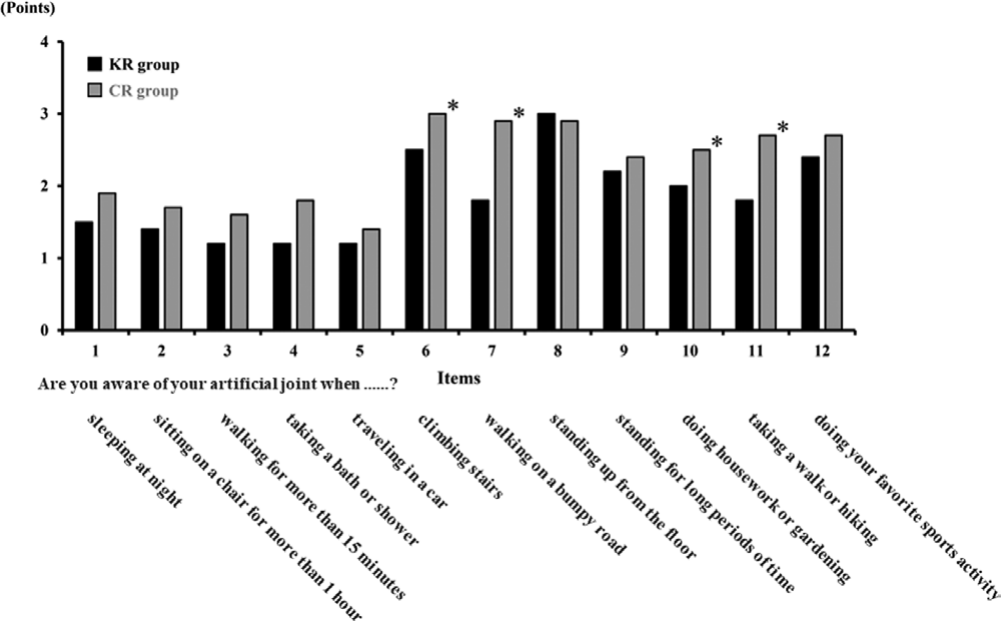
Comparison of patient satisfaction by Forgotten Joint Score-12. The answers were scored as 0 (never), 1 (almost never), 2 (seldom), 3 (sometimes), and 4 (mostly) points. Significance of differences was evaluated by Wilcoxon rank-sum test (*P < .05).

Patient satisfaction evaluated by FJS-12 method demonstrated no significant difference in the total score between KR and CR cases; however, significantly smaller scores in KR group were recorded with respect to the following 4 items: climbing stairs, walking on a bumpy road, doing housework or gardening, and taking a walk or hiking. Thus, the KR implant showed better patient satisfaction compared to the CR, at least in those 4 evaluation items (Fig. 3).

In plain radiographs, there existed no case of loosening and there was no revision case. Also, there have been no cases with obvious complications in both groups during and after surgeries as well. No early failure was observed, and short-term survival rates of both implants were 100%.

## 4. Discussion

The new-type KR knee prosthesis has been designed to recreate natural rolling and gliding mechanisms of knee joint, aiming to restore physiological movement, whilst providing implant stability and pain-free functions. In the current study, patients indicated a higher satisfaction with the KR compared to CR implant, suggesting that TKA procedure using the KR implant could be a good surgical option to improve postoperative outcomes.

Note that since there were different rotational angles of tibial component between the KR and CR implants, ROM technique was used in the KR while tibial component was placed with Akagi line in the CR, in order to determine the above rotational angles. The reasons why the patients’ satisfaction in KR group was higher than that in CR group have been currently unclear. However, the following might be the possible mechanisms: KR femoral implant was intentionally conformed onto the anterior parts of lateral convex in fully extended position of knee joint and KR lateral convex tibial surface might induce screw home movement in knee extension, while roll-back and external rotation of femoral implant could be induced backwards against the tibial component. On the other hand, the CR femoral implant has enhanced high-flex design which safely accommodates up to 155° of flexion while preserving 30% more native bone (Z10011A Project History File on file at Zimmer Biomet). Thus, it is conceivable that this KR implant may introduce more ideal tibial rotation compared with the CR implant.

Padua et al previously reported that the greater ROM could improve the patients’ perception of outcomes, such as their quality of life.^[9]^ Therefore, gaining a higher ROM improvement is likely to enhance patient treatment satisfaction. In the present study, an improvement rate of ROM by the KR implant (108.1%) was substantially higher than that by the CR (104.5%) postoperatively; thus, these results might have contributed to better patient outcome perceptions.

The consequences of implant malalignment include an uneven distribution of intra-articular load and stress, which eventually leads to a revision procedure.^[10]^ Accurate implantation of an individualized TKA prosthesis will effectively reduce the surgical error and avoid the uneven distribution of intra-articular load, thereby stabilizing knee joint, reducing prosthetic loosening, and improving knee functions.^[11–14]^ Kim et al have determined that a longer stability could be achieved when the mechanical axis of femoral component in the sagittal plane ranged between 0° and 3°.^[15]^ Moreover, Gromov et al demonstrated that the optimal clinical results and maximally increased survival period could be obtained when the external rotation angle which was relative to the posterior condylar axis ranged between 2° and 5° in the axial plane.^[16]^

In our cases, the rotation angle of femoral component of the KR implant was determined by 3D templates, in which femoral cutting was performed parallel to transepicondylar axis. Tibial cutting was performed placing 3° as posterior tilt as usual, compared to preoperative original tibial shape. Another point during surgeries was determination of rotational position of the tibial component after confirming that the distal end of femoral component matched anterior part ahead of lateral convex surface of insert using ROM technique, while tibial component in the CR cases was set based on the Akagi line. Currently, the reasons why some clinical outcomes and patient satisfaction items in the KR implant group showed significant differences compared with those in the CR implant group are uncertain. However, it is conceivable that there might be the following reasons: the design of the KR implant could have better contributed to the observations than the CR and ROM technique was useful when the KR prosthesis was used to aim the ideal mounting angle of implant. These findings suggest that the optimal alignment of knee prosthesis could support superior outcomes compared to the CR implant. Whether or not stable long-term results can be achieved should be examined in the future.

This study included several limitations. First, this investigation was a single-centered retrospective study that might have overlooked some information and potential risk factors. Second, this study had a relatively small number of cases with minimum 2 years and more follow-up period. Third, this study included the KR knee data, which could be a source of bias for patient reporting outcomes. Future studies with larger sample size and longer follow-up duration are required to validate long-term significance.

## 5. Conclusions

In the present study, we reported the short-term clinical results of TKA with a new kinematic-retaining type of implant that would be the first time in our country. Some clinical outcomes and patient satisfaction items in this KR implant group showed significant differences compared with those in the CR implant group, although there were no cases of simple radiographic loosening or revision, and their short-term survivorships were 100% in both procedures. The clinical results of the KR prosthesis in the short-term perioperative period were good; especially, postoperative patient satisfaction was significantly higher than that of the CR cases. Our results suggest that this novel prosthetic implant can reproduce physiological kinematics of the knee, recover its functions, and contribute to pain relief after TKA procedure. Further investigations will be needed whether or not our findings are stable in the long-run outcomes.

## Acknowledgments

We would like to thank all participants in the present study as well as Mr. Trevor Ralph for his English language editing.

## Author contributions

**Conceptualization:** Takashige Momose, Yukio Nakamura, Atsushi Sobajima, Masashi Nawata.

**Data curation:** Takashige Momose, Atsushi Sobajima.

**Formal analysis:** Atsushi Sobajima.

**Funding acquisition:** Yukio Nakamura.

**Investigation:** Takashige Momose, Yukio Nakamura.

**Methodology:** Takashige Momose.

**Project administration:** Takashige Momose, Takashi Maeda.

**Resources:** Yukio Nakamura.

**Supervision:** Takashige Momose, Masaki Nakano, Yukio Nakamura.

**Validation:** Takashige Momose, Yukio Nakamura.

**Visualization:** Takashige Momose.

**Writing – original draft:** Takashige Momose, Masaki Nakano, Yukio Nakamura, Susumu Morioka.

**Writing – review & editing:** Takashige Momose, Masaki Nakano, Yukio Nakamura, Takashi Maeda, Susumu Morioka, Masashi Nawata.

## Supplementary Material


