# Supplementary material for: Short-term clinical outcomes of primary total knee arthroplasty with a new-type kinematic retaining implant: A comparison with preexisting cruciate retaining prosthesis
Source: Medicine (Baltimore). 2023 Aug 25;102(34):e34769. doi: 10.1097/MD.0000000000034769 (PMC10470764; doi:10.1097/MD.0000000000034769)
Supplement: Supplementary file 1 [file medi-102-e34769-s001.pptx]

## Slide 1
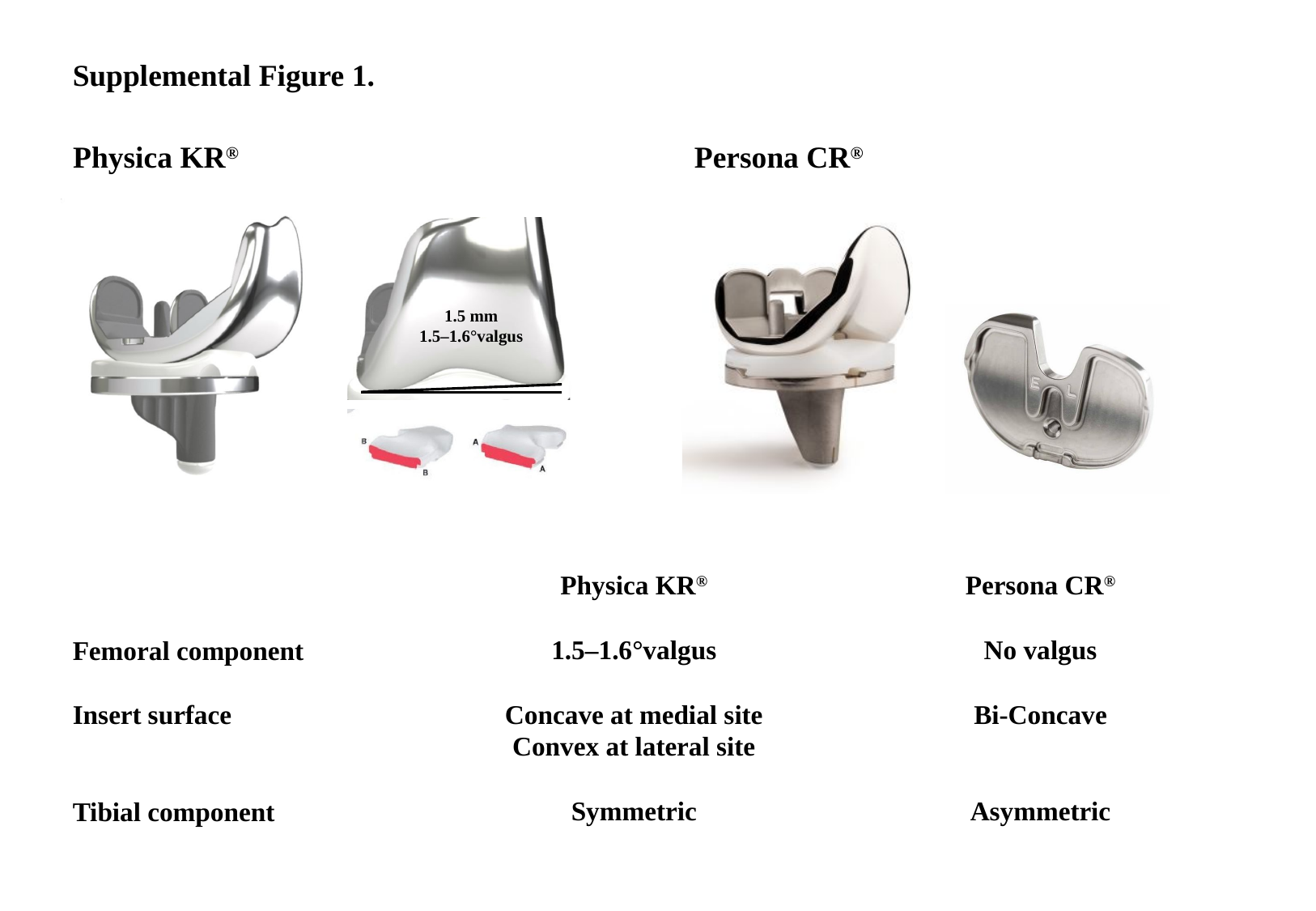

Supplemental Figure 1.
Physica KR®
Persona CR®
1.5 mm
1.5–1.6°valgus
Physica KR®
1.5–1.6°valgus
Concave at medial site
Convex at lateral site
Symmetric
Persona CR®
No valgus
Bi-Concave
Asymmetric
Femoral component
Insert surface
Tibial component
